# Fiscal autonomy of subnational governments and equity in healthcare resource allocation: Evidence from China

**DOI:** 10.3389/fpubh.2022.989625

**Published:** 2022-09-30

**Authors:** Ciran Yang, Dan Cui, Shicheng Yin, Ruonan Wu, Xinfeng Ke, Xiaojun Liu, Ying Yang, Yixuan Sun, Luxinyi Xu, Caixia Teng

**Affiliations:** ^1^Department of Global Health, School of Public Health, Wuhan University, Wuhan, China; ^2^Global Health Institute, Wuhan University, Wuhan, China; ^3^Public Health School, Fujian Medical University, Fuzhou, China

**Keywords:** fiscal autonomy, healthcare resources, equity of allocation, Theil index, econometric methods, mechanism, heterogeneity

## Abstract

**Objectives:**

Promoting equity in healthcare resource allocation (EHRA) has become a critical political agenda of governments at all levels since the ambitious Universal Health Coverage was launched in China in 2009, while the role of an important institutional variable—fiscal autonomy of subnational governments—is often overlooked. The present study was designed to determine the effect of FASG on EHRA and its potential mechanism of action and heterogeneity characteristics to provide empirical support for the research field expansion and relative policies making of EHRA.

**Methods:**

From the start, we utilized the Theil index and the entropy method to calculate the EHRA index of 22 provinces (2011–2020) based on the medical resource data of 287 prefecture-level cities. Furthermore, we used the two-way fixed effects model (FE) to identify and analyze the impact of FASG on EHRA and then used three robustness test strategies and two-stage least squares (2SLS) regression to verify the reliability of the conclusions and deal with potential endogeneity problems, respectively. At last, we extend the baseline regression model and obtain the two-way FE threshold model for conducting heterogeneity analysis, which makes us verify whether the baseline model has nonlinear characteristics.

**Results:**

The static value and the trend of interannual changes in the EHRA values in different provinces are both very different. The regression results of the two-way FE model show that FASG has a significant positive impact on EHRA, and the corresponding estimated coefficient is – 0.0849 (*P* < 0.01). Moreover, this promotion effect can be reflected through two channels: enhancing the intensity of government health expenditure (IGHE) and optimizing the allocation of human resources for health (AHRH). At last, under the different economic and demographic constraints, the impact of FASG on EHRA has nonlinear characteristics, i.e., after crossing a specific threshold of per capita DGP (PGDP) and population density (PD), the promotion effect is reduced until it is not statistically significant, while after crossing a particular threshold of dependency ratio (DR), the promotion effect is further strengthened and still statistically significant.

**Conclusions:**

FASG plays an essential role in promoting EHRA, which shows that subnational governments need to attach great importance to the construction of fiscal capability in the allocation of health care resources, effectively improve the equity of medical and health fiscal expenditures, and promote the sustainable improvement of the level of EHRA.

## Introduction

How to achieve equity in the allocation of health care resources (EHRA) is an important issue facing most countries today (1–3). Equity in health care reflects equal access to public health and health care resources, and plays an essential role in effectively maintaining and enhancing individual health capital (4, 5). There are specific equity differences between and within countries in terms of access, utilization, and quality of medical resources, as well as people's health (6). Therefore, promoting the equity of medical resources and services has been an undeniable governance mission of governments (7–9) and has gradually become the core goal of the medical and health system as well as the requirement for human well-being (10). Since the new round of medical and health system reform was launched in 2009, the Chinese government has regarded the equity of basic public health services and the allocation of medical and health resources as a priority area, and has always been concerned about and committed to promoting the universal coverage and fair allocation of medical and health services and resources (11). On October 25, 2016, the CPC Central Committee and the State Council issued and implemented the “Healthy China 2030” Planning Outline, which pointed out that China should adhere to the principle of fairness and justice, and strive to “promote the equalization of basic public services in the field of health, safeguard the public welfare of basic medical and health services, gradually narrow the differences in basic health services and health levels between urban and rural areas, regions and people, achieve universal health coverage and promote social equity”. Although great progress has been made in promoting EHRA since the implementation of the “Healthy China 2030” strategy, differences in the allocation and quality of medical resources between urban and rural areas, regions, and populations are still apparent obstacles to the construction of “Healthy China”, which has attracted the attention of many researchers (12, 13) and is also a critical enduring issue on the Chinese government's development agenda (14, 15).

The uneven geographic distribution of healthcare resources is one of the most obvious features of healthcare systems around the world, which in turn has been identified as a root cause of regional health inequalities (16). In terms of China, the current allocation of healthcare resources demonstrated significant gaps among 31 provinces and different regions. Healthcare resources, such as doctors, hospitals, and beds, are mainly distributed to economically developed eastern provinces, while the western regions and some poorly developed provinces lack healthcare resources, specifically high-quality ones (3, 17). Judging from the latest provincial 2021 Statistical Yearbook data, there are apparent differences in the stock of healthcare resources in each province and its prefecture-level cities. For example, from the perspective of the number of physicians per 1,000 people, Jiangsu, located on the eastern coast, is 3.16, while Gansu, located inland, is only 2.65. Furthermore, even within the same region, the differences in the distribution of medical resources are still very severe (18). For example, one study pointed out that inequality indexes for technicians and beds in the eastern region continued to increase from 0.016 and 0.072 to 0.028, and 0.116, respectively (2013–2018) (19). In general, financial, material, and human resources are foundation of the healthcare services in China (20). However, the healthcare resource allocation and the supply of health services are systematic projects constrained and affected by many factors (21). For example, some scholars believe that the responsibility of the government health department to allocate medical and health resources is based on the holistic consideration of a region rather than just focusing on the interests of individuals (22), while others also found that the availability of human resources for health, the fiscal utilization capability of subnational governments, and the participation of multiple relevant subjects all have a considerable impact on the allocation of health care resources (23). From the existing literature, researchers focus more on the description and evaluation of the inequality in the allocation of health care resources in a particular country or region (24–26), and less on the comprehensive and profound discussion of the potential influencing factors, especially the lack of attention to some economic and social factors. For instance, fiscal autonomy of subnational government (FASG), as an essential institutional variable that can effectively reflect the subnational government's capability to respond to events and the efficiency of public resource allocation as well as governance performance (27, 28) has been intentionally or not ignored in the discussion and research on the topic of EHRA. Hence, much uncertainty still exists about the relationship between FASG and EHRA. Likewise, the mechanisms that underpin FASG impacting EHRA are not fully understood, which are deficiencies and regrets of current studies.

Based on the discussion above, this study, therefore, set out to assess the impact of FASG on EHRA. In our analysis, we first calculate the EHRA index of 22 provinces in China (2011–2020) since the medical and health system reform was launched in 2009. Next, with the help of econometric methods, we will try to identify and analyze the impact of FASG on EHRA and its potential mechanism as well as the realistic heterogeneity, allowing us to obtain empirical support and improvement ideas conducive to promoting the EHRA.

## Methods

### Data sources

Our empirical analysis is mainly based on panel data from 22 provinces in China in the period 2011–2020. Because of our research purpose focusing on EHRAs within provincial-level government jurisdictions and the severe lack of consistent data in the period covered, our sample does not include four municipalities (Beijing, Tianjin, Shanghai, and Chongqing), as well as Tibet, Qinghai, Yunnan, and Xinjiang. In our study, the relevant data used to calculate the EHRA index, including the number of various types of licensed medical institutions, the number of beds in medical institutions, the number of licensed doctors (including assistant doctors), and the number of registered nurses, are mainly derived from the Statistical Yearbooks of 287 prefecture-level cities in 22 provinces. To ensure the consistency of the statistical caliber of corresponding data, we spent much time checking and supplementing the data by consulting many statistical bulletins on prefecture-level cities' economic and social development. In addition, it should be noted that due to the lack of data in some years of individual prefecture-level cities, we use the linear interpolation method and the geometric mean method to fill in the data according to the specific situation. In this paper, provincial-level data are collected in a variety of ways. Total health expenditure, number of licensed medical institutions, number of licensed doctors (including assistant doctors), and number of registered nurses are from the China Health Statistical Yearbook (2012–2021). The dependency ratio (DR) and the illiteracy rate (IR) of population aged 15 and above are from the China Population and Employment Statistical Yearbook (2012–2021). Other data, such as the number of permanent residents, highway mileage, government fiscal revenue and expenditure, and some deflator indices, are from the China Statistical Yearbook and the provincial statistical yearbooks (2012–2021).

### Dependent variable: EHRA index

Healthcare resources were measured by considering two key dimensions: material resources and human resources, including the number of medical institutions, the number of beds in medical institutions, the number of licensed doctors (including assistant doctors), and the number of registered nurses. It should be note that the statistical caliber of “medical institutions” in the above four indicators involved all medical and health institutions that have obtained the legal grade certificate of the health administrative department, including hospitals, grass-roots medical and health institutions and professional public health institutions. The Advantage of this approach for measurement can make EHRA indexes capture across all jurisdictions in different provinces and contain more comprehensive medical resource information. Furthermore, we averaged the above four indicators in combination with the administrative area of prefecture-level cities and the number of permanent residents to obtain the number of medical institutions per square kilometer, the number of beds in medical and health institutions per 1000 people, the number of licensed physicians (including assistant physicians) per 1000 people and the number of registered nurses per 1000 people, to eliminate the impact of geographical scope and population size.

Gini coefficient, coefficient of variation, and Theil index are widely used to measure the level of EHRA (29). Referring to the existing literature (19, 30), we use the Theil index to measure the degree of equity of the four indicators mentioned above, respectively, and the calculation formula is as follows:


(1) $$\begin{array}{l} T=\frac{1}{n}\sum {}_{i=1}^{n}\frac{y_{i}}{y}log\left(\frac{y_{i}}{y}\right) \end{array}$$

Where *T* is the Theil index to measure the degree of equity of different indicators, *y*_*i*_ and $\overset{\bar{}}{y},$ respectively represent the number of specific medical resources and the average number of such medical resources in various cities, and *n* is the number of prefecture-level cities under the jurisdiction of a province. The larger the *T* value, the lower the EHRA level, and vice versa. Meanwhile, we also, respectively used the coefficient of variation and the Gini coefficient as alternative measurement methods and calculated the corresponding EHRA index for the robustness test in the following. The specific calculation formulas can be referred to in relevant literature (31, 32). After measuring the above four medical resource indicators according to the Theil index, we used the entropy method (33) to assign weight to the four indicators objectively. Finally, we synthesized them into the EHRA index to characterize the degree of inequality in the healthcare resource allocation in a particular area. The smaller the EHRA index value of a province, the higher the equity level, and vice versa. Theil index, coefficient of variation, and Gini coefficient were calculated using the R Version 4.1.2 “REAT” package.

### Independent variable: FASG

FASG not only reflects the fiscal ability and operation of subnational governments but is also a critical factor affecting the scope and standard of public services provided by subnational governments to the public (34, 35). Since implementing the tax-sharing reform in 1994, China has formed a fiscal and tax allocation pattern with apparent characteristics between the central government and subnational governments (36, 37). The appropriate measurement of FASG is a difficult work given the complexity of intergovernmental relations, and needs a method suitable for the Chinese situation. To this aim, we calculate FASG by the ratio of general budget revenue to general budget expenditure to represent the fiscal pressure of the subnational government based on the existing research literatures (38, 39). The larger the FASG value, the higher the proportion of the subnational government's fiscal revenue, i.e., the weaker the subnational government's dependence on the central government's transfer payments, meaning broader fiscal revenue resources and a more flexible expenditure structure for giving subnational governments sufficient motivation to provide better public services (including healthcare recourses). Conversely, less the FASG value suggests that subnational governments are more dependent on transfer payments from the central government, which will limit, to some extent, the subnational government's ability to provide public services (40, 41).

### Mediator variable: IGHE and AHRH

Previous studies have shown that sufficient fiscal funds can make subnational governments more capable of formulating a series of systematic and scientific policy implementation plans with more sustainable public health expenditures (42). Moreover, government resources, including fiscal funds, are needed for direct support to address the estimated deficit in the health workforce (43). Therefore, to reveal the potential mechanism of FASG affecting EHRA while taking into account the difficulties in identifying and measuring some other potential mediating variables, we only select the intensity of government health expenditure (IGHE) and the allocation of human resources for health (AHRH) as mediator variables for subsequent empirical tests. It should be noted that IGHE is measured by the proportion of government health expenditure to the total social health expenditure, and AHRH is measured by the sum of the number of licensed doctors (including assistant doctors) and registered nurses at the provincial level.

### Control variables and threshold variables

To minimize the estimation error caused by the omission of variables in the subsequent model regression process, we consider a series of factors related to economy, population, infrastructure, and culture that may affect the dependent variables, including per capita GDP (PGDP) indicating the level of regional economic development (treated by GDP deflator), PD reflecting the size and degree of agglomeration of the regional population, per capita highway mileage indicating infrastructure and traffic conditions, DR representing the burden of supporting the elderly and young children, and IR of population reflecting the degree of regional social and cultural development. However, Due to the inherent complexity of socioeconomic factors, it is not easy to measure and control some variables, such as the behavioral preferences of subnational government officials with primary responsibility and the public's actual needs for healthcare resources, which may influence the outcome variable, EHRA. Hence, to overcome the potential limitations of omitted variables, we employ a series of econometric analysis techniques to discuss and correct for biases in subsequent analyses. In addition, the corresponding control variables will be analyzed one by one as threshold variables to identify the threshold characteristics of FASG affecting EHRA under different constraints. Table 1 provides the measurement methods and descriptive statistics of different variables.

**Table 1:** Variable description and statistics.

**Variables**	**Description**	**Mean**	**SD**
THEIL	EHRA index calculated based on Theil index and entropy method	0.0796	0.0541
CV	EHRA index calculated based on coefficient of variation and entropy method	0.0895	0.0288
GINI	EHRA index calculated based on Gini coefficient and entropy method	0.1883	0.0494
IGHE	Proportion of government health expenditure in total social health expenditure	0.3058	0.0632
AHRH	The sum of the number of licensed doctors (including assistant doctors) and registered nurses	12.2909	0.6357
FASG	The ratio of general budget revenue to general budget expenditure of subnational government	0.4820	0.1597
PGDP	GDP per capita calculated based on the permanent population	10.6344	0.3669
PD	Number of permanent residents per square kilometer	5.4740	0.8721
TA	Number of highway meters per capita calculated based on the permanent population	3.6242	1.4012
DR	The ratio of the total number of children and the elderly to the number of labor force population	0.3811	0.0705
IR	Illiteracy rate of population aged 15 and above	0.0415	0.0195

Note: SD, standard deviation; IGHE, intensity of government health expenditure; AHRH, allocation of human resources for health; FASG, fiscal autonomy of subnational government; PGDP, per capita GDP; PD, population density; TA, traffic accessibility; DR, dependency ratio; IR, illiteracy rate. In order to mitigate the influence of heteroskedasticity and multicollinearity on the regression results, we perform natural logarithmic transformations on AHRH, PGDP, and PD.

### Empirical strategy

The econometric analysis in this study will be carried out in the following order: baseline regression, mechanism analysis, and heterogeneity analysis.

Firstly, we constructed the two-way fixed effects model, a default method for estimating causal effects, as the beginning of empirical research based on panel data (44). The regression model is specified as follows.


(2) $$\begin{array}{l} THEIL_{it}=\alpha +\beta FASG_{it}+\sum {}_{l=1}^{c}\delta_{l}X_{lit}+\varphi_{i}+\omega_{t}+\varepsilon_{it} \end{array}$$

where *THEIL*_*it*_ is the dependent variable we care about, which is the EHRA index calculated by using the Theil index and the entropy method in combination (*i* represents the provinces, *t* represents the year), *FASG*_*it*_ is the key independent variable, α is the constant, *X*_*lit*_ is a set of control variables, φ_*i*_ and ω_*t*_ are unit and time fixed effects representing these unit-specific and time-specific unobserved confounders that are common causes of the dependent variable, and ε_*it*_ is a stochastic disturbance term.

Second, to identify the potential influencing mechanism, we replaced the dependent variable of the baseline model with *IGHE*_*it*_ and *AHRH*_*it*_, respectively. Therefore, we can rewrite Eq. (2) as follows:


(3) $$\begin{array}{l} \begin{array}{l} IGHE_{it}=\alpha +\beta FASG_{it}+\sum {}_{l=1}^{c}\delta_{l}X_{lit}+\varphi_{i}+\omega_{t}+\varepsilon_{it} \end{array} \end{array}$$

(4) $$\begin{array}{l} \begin{array}{l} AHRH_{it}=\alpha +\beta FASG_{it}+\sum {}_{l=1}^{c}\delta_{l}X_{lit}+\varphi_{i}+\omega_{t}+\varepsilon_{it} \end{array} \end{array}$$

Third, to investigate whether each control variable's restriction degree will cause the heterogeneous performance of the impact of FASG on the EHRA index, we constructed a threshold panel regression model of different control variables on the EHRA index on the method proposed by Hansen (45). Eq. (2) is rewritten to be a two-way FE threshold model as follows:


(5) $$\begin{array}{l} \;THEIL_{it}=\alpha +\beta_{1}FASG_{it}I\left(X_{k}\leq \gamma \right)+\beta_{2}FASG_{it}I\left(X_{k}>\gamma \right)\\ +\sum {}_{l=1,l\neq k}^{c-1}\delta_{l}X_{lit}+\varphi_{i}+\omega_{t}+\varepsilon_{it} \end{array}$$

In Eq. (5), the main variables have the same meanings as those in Eq. (2). *X*_*k*_ is the threshold variable, the *k*th variable selected from the set of control variables in Eq. (2). γ is the estimated threshold value, while *I*(·) is the indicator function. In the later model estimation, we need to test the significance and authenticity of the threshold effect. If both tests pass simultaneously, we will subsequently estimate the double-threshold or multi-threshold effects. Due to the limited space of the paper, the specific econometric equations are not shown in detail. Econometric analysis was performed in this part using Stata/MP, Version 16(StataCorp LP, College Station, TX, USA).

## Results

### Calculation results of EHRA index for each province

Table 2 provides the results obtained from the preliminary calculation of the EHRA index for each province by comprehensively using the Theil index and entropy method. In general, affected by factors such as the level of economic and social development and differences in natural and geographical conditions, the cross-sectional values and interannual changes of the EHRA index in each province are different. On the one hand, from the perspective of interannual trend, Zhejiang, Hubei, and Liaoning have slight changes, with rangeability of only 0.0047, 0.0117, and 0.0135, respectively, while Gansu, Fujian, and Ningxia have sharp interannual fluctuations, with rangeability of 0.0529, 0.0523, and 0.0378, respectively. On the other hand, from the perspective of annual mean value, the EHRA index mean values of Jiangsu, Shandong and Zhejiang rank among the top three, while the EHRA index mean values of Inner Mongolia, Gansu, Sichuan and Guangdong all exceed 0.1, which shows that the healthcare resource allocation within the jurisdiction varies greatly.

**Table 2:** Calculation results of EHRA Index in 22 provinces of China (2011–2020).

**Province**	**Ranking**	**Mean**	**SD**	**Min**	**Max**
Jiangsu	1	0.0186	0.0053	0.0147	0.0334
Shandong	2	0.0313	0.0053	0.0243	0.0415
Zhejiang	3	0.0344	0.0014	0.0311	0.0358
Hunan	4	0.0345	0.0062	0.0223	0.0413
Jiangxi	5	0.0361	0.0053	0.0262	0.0429
Anhui	6	0.0412	0.0125	0.0255	0.0624
Jilin	7	0.0505	0.0055	0.0450	0.0658
Guizhou	8	0.0520	0.0091	0.0434	0.0717
Shanxi	9	0.0545	0.0051	0.0411	0.0601
Liaoning	10	0.0548	0.0044	0.0475	0.0610
Henan	11	0.0574	0.0056	0.0497	0.0716
Guangxi	12	0.0685	0.0100	0.0528	0.0866
Ningxia	13	0.0729	0.0120	0.0527	0.0905
Hebei	14	0.0754	0.0104	0.0631	0.0937
Shaanxi	15	0.0848	0.0083	0.0770	0.1046
Hubei	16	0.0934	0.0040	0.0881	0.0998
Fujian	17	0.0944	0.0185	0.0706	0.1229
Heilongjiang	18	0.0948	0.0045	0.0893	0.1031
Guangdong	19	0.1253	0.0108	0.1103	0.1444
Sichuan	20	0.1442	0.0099	0.1347	0.1720
Gansu	21	0.1950	0.0156	0.1629	0.2158
Inner Mongolia	22	0.2376	0.0058	0.2301	0.2467

Note: SD, standard deviation. The ranking of each province is determined according to the annual average of the EHRA Index.

### Panel unit root test

To avoid the possible pseudo-regression phenomenon in the modeling process, we test each variable one by one to determine whether they have unit roots before econometric analysis. Referring to the practice of prior scholars (46, 47), we choose to use four traditional unit root test techniques, LLC, IPS, Fisher ADF, and Fisher PP, to identify the stationarity of each variable. The null hypothesis of the four testing techniques is H0: The variable has a unit root. Nonetheless, due to the differences in the principle and premise of the unit root test, different test methods may draw different conclusions when testing the variable data. Hence, according to the principle of the minority obeying the majority, we comprehensively judge whether the data is stationary according to the four test results to improve the power and reliability of the test. As shown in Table 3, THEIL, CV, GINI, PGDP, and PD failed to reject the null hypothesis of the IPS test, and FASG failed to reject the null hypothesis of the Fisher-ADF test, and PHR failed to reject the null hypothesis of Fisher-PP test. But taken together, most panel unit root tests reject the null hypothesis at the 1% significance level (or 5% significance level). Based on the unit root test results, we believe that all the variables included in the model analysis have good stationarity and meet the requirements of subsequent econometric model analysis.

**Table 3:** Panel unit root test.

**Variables**	**Type (*c*, *t*, *l*)**	**LLC**	**IPS**	**Fisher-ADF**	**Fisher-PP**	**Smooth**
THEIL	(1,0,1)	– 3.0723 [0.0011]	1.5428 [0.9386]	2.6129 [0.0045]	2.1218 [0.0169]	YES
CV	(1,0,1)	– 6.2910 [0.0000]	– 0.7739 [0.2195]	4.3991 [0.0000]	6.2675 [0.0000]	YES
GINI	(1,0,1)	– 6.5694 [0.0000]	– 0.6769 [0.2492]	5.4675 [0.0000]	7.4709 [0.0000]	YES
IGHE	(1,1,1)	– 12.7425 [0.0000]	– 3.7924 [0.0001]	16.5861 [0.0000]	4.8814 [0.0000]	YES
AHRH	(1,1,1)	– 16.5825 [0.0000]	– 4.8315 [0.0000]	12.4263 [0.0000]	4.8800 [0.0000]	YES
FASG	(1,1,1)	– 8.4136 [0.0000]	– 1.6540 [0.0491]	0.8098 [0.2090]	6.4747 [0.0000]	YES
PGDP	(1,0,1)	– 1.7818 [0.0374]	1.4939 [0.9324]	3.9335 [0.0000]	3.7478 [0.0001]	YES
PD	(1,0,1)	– 3.2163 [0.0006]	2.2455 [0.9876]	3.9560 [0.0000]	19.5012 [0.0000]	YES
TA	(1,1,1)	– 6.7772 [0.0000]	– 1.6254 [0.0520]	6.1151 [0.0000]	0.7867 [0.2157]	YES
DR	(1,1,1)	– 11.0506 [0.0000]	– 3.2832 [0.0005]	4.8417 [0.0000]	5.4105 [0.0000]	YES
IR	(1,0,1)	– 6.0050 [0.0000]	– 3.1564 [0.0008]	10.0655 [0.0000]	2.7043 [0.0034]	YES

Note: In the test type, *c* the constant term, *t* the trend term, and *l* the lag order. Values outside square brackets are asymptotic statistics, and values inside square brackets are the corresponding *p*-values. In the Fisher-ADF test and Fisher-PP test, the statistics we report are the corrected inverse χ^2^ statistics and their *p*-values.

### Baseline regression results

In our analysis, we also take into consideration whether there is multicollinearity between the independent variables included in the model, so we make a statistical test before estimating the baseline regression model. The test results show that the maximum variance inflation factor (VIF) is 7.70, the minimum VIF is 1.50, and the mean VIF is 4.10, which is less than the critical value 10. Hence, we believe there is no need to worry about the potential multicollinearity between variables.

When choosing a suitable model for regression, we first compare the pooled regression model and the fixed effects model (FE). In the case of considering the cross-sectional correlation of the data, the value of test F statistic of the province dummy variable is 6000.35 (*P* < 0.01), and the test result rejects the null hypothesis that there are no province fixed effects, which indicates that the fixed effects model should be selected for regression. We also compared the random effects model (RE) and FE, and the value of Hausman test statistics was 14.07 (*P* < 0.05), which still supported the acceptance of FE. In addition, this conclusion is still valid under the premise of considering and dealing with the three problems of heteroscedasticity, autocorrelation, and cross-sectional correlation, which shows that it is highly applicable to choose FE for regression.

Because the relevant tests in the model selection process show that ε_*it*_ has three major problems, including heteroscedasticity, autocorrelation, and cross-sectional correlation, we dealt with these problems in model regression and reported the estimated results of univariate two-way FE regression, one-way FE regression with control variables and complete two-way FE regression, respectively in Columns (1) to (3) in Table 4. From the results of Columns (1) to (3), the estimated coefficients of FASG in different models are significantly negative at the 1% level. Columns (4), (5), and (6), respectively report the estimated results of two-way FE regression, RE regression, and pooled regression for comparison. It is interesting to note that in Columns (3), (4), and (6), the estimated coefficients of the core independent variable FASG and the control variable are all equal, and the estimated results of the core independent variable jointly show that FASG can significantly and negatively impact THEIL (β = – 0.0849, *P* < 0.01). Although the estimated results of columns (3), (4), and (6) are very close, we prefer to believe the estimated results of Column (3), i.e., the results of two-way FE regression after dealing with the three major problems of ε_*it*_. In the analysis based on baseline regression, we found that a more effective state of FASG is associated with a better EHRA, i.e., a more balanced and equal healthcare resource allocation. Meanwhile, the estimated coefficients of the control variables in Column (3) are all consistent with the theoretical expectations. Specifically, PGDP, DR, and TA all negatively impact THEIL, but only TA passed the significance test. PD and IR showed a positive effect on THEIL, but they were not statistically significant.

**Table 4:** Baseline regression results of the impact of FASG on EHRA.

**Variables**	**(1)**	**(2)**	**(3)**	**(4)**	**(5)**	**(6)**
	**FE_1**	**FE_2**	**FE_3**	**FE_4**	**RE**	**OLS**
FASG	– 0.0795^a^	– 0.0889^a^	– 0.0849^a^	– 0.0849^b^	– 0.0652^b^	– 0.0849^a^
	(0.0197)	(0.0126)	(0.0156)	(0.0368)	(0.0313)	(0.0225)
PGDP		– 0.0043	– 0.0010	– 0.0010	0.0050	– 0.0010
		(0.0036)	(0.0146)	(0.0392)	(0.0285)	(0.0236)
PD		0.0176	0.0123	0.0123	– 0.0483^a^	0.0123
		(0.0142)	(0.0119)	(0.0547)	(0.0138)	(0.0308)
TA		– 0.0112^a^	– 0.0129^a^	– 0.0129^b^	– 0.0151^a^	– 0.0129^a^
		(0.0013)	(0.0007)	(0.0051)	(0.0056)	(0.0029)
DR		– 0.0470	– 0.0980	– 0.0980^c^	– 0.1109^b^	– 0.0980^b^
		(0.0309)	(0.0568)	(0.0477)	(0.0559)	(0.0399)
IR		0.0841	0.1913	0.1913	0.2005	0.1913
		(0.1518)	(0.1410)	(0.2435)	(0.2294)	(0.1890)
Constant	0.1257^a^	0.1265	0.1391	0.1391	0.4091	0.3671
	(0.0099)	(0.0887)	(0.1805)	(0.5488)	(0.2817)	(0.2824)
Province FE	YES	YES	YES	YES	NO	YES
Time FE	YES	NO	YES	YES	YES	YES
R^2^	0.1253	0.2191	0.2443	0.4594	0.4484	0.9751
Observations	220	220	220	220	220	220

Note: Standard errors in parentheses, where Columns (1) to (3) report the Driscoll-Kraay standard errors, which are used to solve the three major problems of heteroskedasticity, autocorrelation, and cross-section correlation, Columns (4) to (6) report the cluster-robust standard errors, which are used to solve the two major problems of heteroskedasticity and autocorrelation. The R^2^ reported in Columns (1) to (4), (5), and (6) are Within R^2^, Overall R^2^, and Adj.R^2^, respectively. ^a^*P* < 0.01, ^b^*P* < 0.05, ^c^*P* < 0.10.

### Endogenous treatment

Although the baseline regression model controls many potentially related provincial socioeconomic variables and unobservable provincial heterogeneity factors that do not change over time, it still may have endogeneity problems caused by the omission of variables, which may lead to the bias of regression results. Therefore, we take the lag phase of FASG as the instrumental variable (IV) of FASG to run two-stage least squares (2SLS) regression. In Table 5, we present the estimated results of 2SLS regression. Clearly, the test result in the first-stage regression shows that the correlation coefficient between IV and FASG is very statistically significant, and the F-statistic is much larger than the empirical value 10, indicating that IV has a strong correlation with the potential endogenous independent variable (FASG). Some observations similar to baseline regression can be made in the second-stage regression. Although the estimated coefficient of FASG has increased, the negative impact of FASG on Theil is still significant at the 1% level. After running 2SLS regression and dealing with possible endogenous problems, we found that the estimated results based on baseline regression were further confirmed, i.e., FASG showed a significant promotion effect on EHRA.

**Table 5:** 2SLS regression based on instrumental variable.

**Variables**	**(1)**	**(2)**
	**First stage**	**Second stage**
FASG_t − 1_	0.4694^a^	– 0.2027^a^
	(0.0736)	(0.0613)
Constant	– 2.1434^b^	– 0.6466
	(0.8760)	(0.4217)
Control variables	YES	YES
Province FE	YES	YES
Time FE	YES	YES
Overall R^2^	0.7744	0.5254
F/Wald χ^2^	46.27	17107.82
Observations	198	198

Note: Standard errors in parentheses. ^a^ *P* < 0.01, ^b^ *P* < 0.05, ^c^ *P* < 0.10. The test statistics reported in Columns (1) 2SLS, two-stage least squares and (2) FASGt-1, the one lag phase of FASG are F statistic and Wald statistic, respectively. Cragg-Donald Wald F statistic is 40.682 (*P* < 0.01).

### Robustness check

To ensure the reliability of the conclusion, we will use three robustness test strategies to re-estimate the two-way FE regression model established above in this part. At first, replace the explained variable, i.e., use the new EHRA index constructed based on the coefficient of variation and Gini coefficient, both weighted by the entropy method as the dependent variables for regression. Secondly, eliminate some samples, i.e., re-estimate the model after excluding the data of 2020, because there may be statistical fluctuations or even anomalies in some data on economic, social, medical, and health in 2020 due to the impact of COVID-19, which may have an impact on the estimated results. Finally, add additional control variables, i.e., add additional provincial control variables into the model, including the development level of the service industry (proportion of the added value of the tertiary industry in GDP), the level of subnational governments' public service (proportion of general public service expenditure in total fiscal expenditure) and the intensity of educational investment (proportion of education expenditure in total fiscal expenditure), for re-estimating the baseline model. From the regression results, as shown in Table 6, the estimated coefficients of FASG in all columns are negative and statistically significant (*P* < 0.01), especially the estimated results in columns (3) and (4) are very close to the baseline regression results, which reconfirms the positive effect of FASG on EHRA and indicates the estimation results in this study are robust.

**Table 6:** Robustness test.

**Variables**	**(1)**	**(2)**	**(3)**	**(4)**
	**CV**	**GINI**	**TIME**	**CONTROL**
FASG	– 0.0478^a^	– 0.0976^a^	– 0.0924^a^	– 0.0922^a^
	(0.0115)	(0.0166)	(0.0204)	(0.0108)
Constant	0.6949^c^	1.1615^b^	0.1913	0.2162
	(0.3364)	(0.4360)	(0.1489)	(0.3236)
Control variables	YES	YES	YES	YES
Province FE	YES	YES	YES	YES
Time FE	YES	YES	YES	YES
Overall R^2^	0.3218	0.4058	0.2933	0.2798
Observations	220	220	198	220

Note: The Driscoll-Kraay standard errors in parentheses. ^a^*P* < 0.01, ^b^*P* < 0.05, ^c^*P* < 0.10.

### Mechanism analysis

So far we have shown that FASG, to some extent, has shaped the EHRA. We now turn to test whether the impact of FASG upon EHRA work through some channels. Some studies have found that governments often make arrangements for fiscal expenditures in the medical and health field based on their fiscal capability (48, 49) and the regional AHRH is a key factor affecting the equity and availability of medical resources under the leading role of government investment (50, 51). Therefore, to further explore how FASG affects EHRA, we will try to analyze the mechanism with the intensity of government health expenditure (IGHE) as one channel and the allocation of human resources for health (AHRH) as another channel. In this part, we will use Eq. (3) and Eq. (4) (IGHE and AHRH as dependent variables) for further analysis. Columns (1) and (2) in Table 7 report the estimated results of the above two equations, respectively. FASG has a significant positive impact on both IGHE and AHRH, which indicates that the stronger FASG is, the more favorable it is for the subnational government to increase fiscal expenditure in the health field and expand the supply of human resources for health, thus facilitating the EHRA.

**Table 7:** Mechanism analysis: IGHE and AHRH as two channels.

**Variables**	**(1)**	**(2)**
	**IGHE**	**AHRH**
FASG	0.0474^b^	0.1079^a^
	(0.0189)	(0.0238)
Constant	2.3867^a^	– 2.6754
	(0.5694)	(1.6062)
Control variables	YES	YES
Province FE	YES	YES
Time FE	YES	YES
Overall R^2^	0.5937	0.9769
Observations	198	220

Note: The Driscoll-Kraay standard errors in parentheses. ^a^*P* < 0.01, ^b^*P* < 0.05, ^c^*P* < 0.10. Due to the missing data on total social health expenditure in 2020, there are only 198 observations in Column (1).

### Heterogeneity analysis

**Figure 1:**
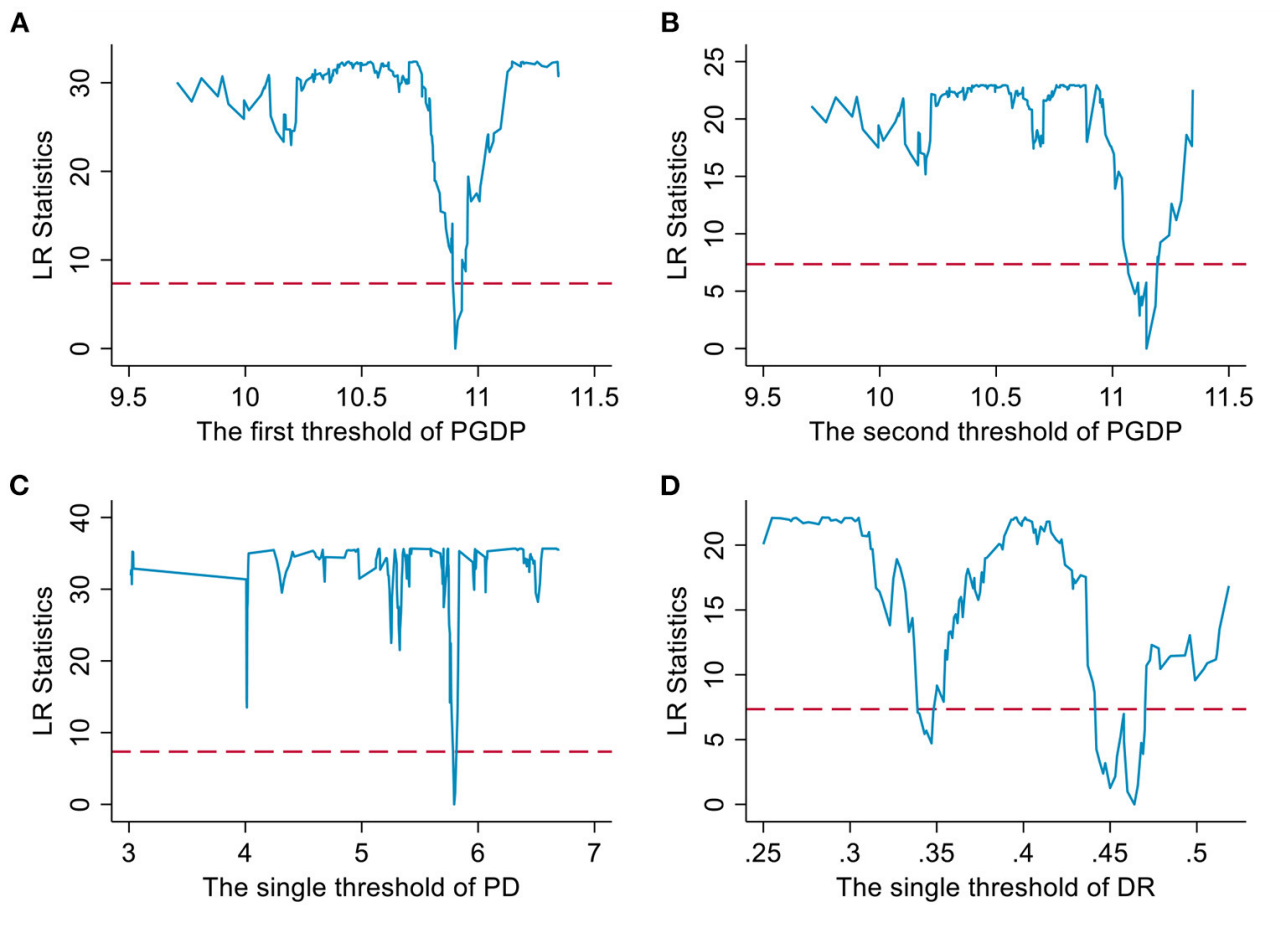
Threshold effect tests for threshold variables. **(A)** and **(B)** are threshold effect tests for PGDP. **(C)** is threshold effect test for PD. **(D)** is threshold effect test for DR.

In our view, the impact of FASG on EHRA may be restricted by different economic and social development conditions, which are likely to be heterogeneous. In this part, we will use Eq. (5) to conduct regression one by one with different control variables as threshold variables to verify whether the model has nonlinear characteristics. Hansen's threshold model determines the threshold value based on the minimization of the residual squared sum, i.e., the closer the estimated γ in the regression is to the true threshold value, the smaller the residual squared sum should be (see the lowest point of the broken line in Figure 1). The next step is to test the authenticity of the estimated gamma using the maximum likelihood ratio statistic (LR) given by Hansen. If the LR of the estimated γ is lower than the empirical test value at the 5% statistical significance level, 7.35, the authenticity of the estimated threshold value can be guaranteed (see the red horizontal dashed line in Figure 1). So we first performed the threshold effect test to determine whether there was a threshold effect and how many threshold values were present and then used the bootstrap method (52) to obtain the statistic *p*-value for the test of the corresponding threshold. The estimated results shown in Table 8 and the graph of LR statistics estimated based on the bootstrap method shown in Figure 1 indicate that PGDP, PD, and DR all passed the threshold test. According to the analysis of Column (1), there are two threshold values of PGDP, which are 10.9019 and 11.1462. When PGDP is lower than 10.9019, FASG has a strong and significant promotion effect on EHRA, with an estimated coefficient of – 0.0576 (*P* < 0.01). When PGDP is between 10.9019, and 11.1462, the estimated coefficient of FASG on EHRA decreases to – 0.0369 due to some economic constraints (*P* < 0.10). However, after PGDP exceeded 11.1462, the second threshold value, the effect mentioned above was further reduced and was not statistically significant, and the corresponding estimated coefficient was reduced to – 0.0165 (*P* = 0.48). The results in Column (2) show that there is a single threshold value of PD. When PD is lower than 5.7944, FASG has a significant promotion effect on EHRA, with an estimated coefficient of – 0.0750. When PD exceeded 11.1462, the estimated coefficient of FASG on EHRA decreased to – 0.0286. The former is statistically significant (*P* < 0.01), while the latter is not (*P* = 0.23), which indicates that the over-concentration of the population will inhibit the promotion effect of FASG on EHRA. In addition, DR also has a single threshold value shown in Column (3). When DR is lower than 0.4640, FASG has a significant promotion effect on EHRA, with an estimated coefficient of – 0.0729 (*P* < 0.01); and different from the threshold characteristics of PGDP and PD mentioned above, when DR exceeds 0.4640, the threshold value, the estimated coefficient increases to – 0.0981 and remains statistically significant (*P* < 0.01), which indicates that the increase of DR will force subnational governments to pay more attention to the equal allocation of medical and health resources and amplify the promotion effect of FASG on EHRA.

**Table 8:** Heterogeneity analysis: two-way FE threshold regression.

**Variables**	**(1)**	**(2)**	**(3)**
	**PGDP**	**PD**	**DR**
γ_1_	10.9019	5.7944	0.4640
γ_2_	11.1462		
FASG·I(q ≤ γ_1_)	– 0.0576^a^	– 0.0750^a^	– 0.0729^a^
	(0.0212)	(0.0222)	(0.0244)
FASG·I(q>γ_1_)		– 0.0286	– 0.0981^a^
		(0.0240)	(0.0246)
FASG·I(γ_1_ <q ≤ γ_2_)	– 0.0369^c^		
	(0.0222)		
FASG·I(q>γ_2_)	– 0.0165		
	(0.0232)		
F_1_ value of one threshold test	29.77^b^	38.39^b^	23.84^c^
	[0.0333]	[0.0333]	[0.0733]
F_2_ value of two threshold tests	24.85^b^		
	[0.0267]		
Control variables	YES	YES	YES
Province FE	YES	YES	YES
Time FE	YES	YES	YES
Overall R^2^	0.5071	0.2258	0.4961
Observations	220	220	220

Note: Values in parentheses are standard errors, and values in square brackets are *p*-values. ^a^*P* < 0.01, ^b^*P* < 0.05, ^c^*P* < 0.10.

## Discussion

In this study, we first calculated the EHRA index of 22 provinces (2011–2020) based on the medical resource data of 287 prefecture-level cities collected by hand and then used econometric methods to conduct a more standardized, rigorous, and robust analysis of the impact of FASG on EHRA, including mechanism analysis and heterogeneity analysis, which allowed us to obtain a series of interesting findings in the end.

From the late 1990s to the beginning of the 20th century, China's medical and health care industry entered the track of market-oriented development. Although this change has led to an increase in the overall amount of medical and health resources, it has also spawned the problem of excessive concentration of medical and health resources in economically developed areas, which in turn has led to a severe contradiction between the people's need for a better and healthy life and the unbalanced and insufficient development of medical and health resources (53–55). However, since 2009, the Chinese government has introduced and implemented a large number of reform policies involving the medical and health system, which have alleviated the imbalance in medical and health resources to a certain extent. From the calculation results mentioned above of the EHRA index in 22 provinces, it can be seen that most provinces have shown an improving trend in interannual changes, while some provinces have experienced an increase in the inequality of medical and health resources. Besides, there is a significant gap in the level of EHRA in provinces with different economic development (56), including some provinces (e.g., Zhejiang and Guangdong) that are in developed coastal areas of China's economy. These circumstances show that, subject to the impact of various factors, the contradiction of interprovincial imbalance of medical and health resources is still prominent and has specific and complex manifestations, as well as conducting targeted research and exploring suitable measures to achieve the optimal allocation of medical and health resources has become more and more necessary.

It is a particular challenge for subnational governments to promote the equity and efficiency of distributing medical and health resources restricted and affected by different economic and social development factors (57). The most apparent and important finding to emerge from the analysis is to confirm that the FASG has a promotion effect on EHRA, i.e., the higher the FASG, the more capable subnational governments are in improving the level of equity of the allocation of medical and health resources in their jurisdiction. In most cases, the decentralization of health policy responsibilities is quite common, even in unitary states. Especially in China, for a long time, under the impact of the GDP growth-oriented political performance appraisal system, Chinese subnational government officials do not need to be responsible for the equity and efficiency of the allocation of medical and health resources and services within their jurisdiction (58), which has led to government fiscal expenditures often have economic preferences. Nevertheless, to our pleasure, with the change of the government performance appraisal system and the in-depth advancement of medical and health system reform, we have observed in recent years some positive changes in government policies that are conducive to the removal of these distortions. Nowadays, subnational governments have abandoned blind pursuits of economic indicators and their fiscal expenditures are more used in livelihood fields (59), including medical and health undertakings, which can drive the construction of medical infrastructure and the training of workers for health in remote and underdeveloped areas in different provinces and reduce the polarization gap in the allocation of medical and health resources in different provinces.

As discussed above, to see how FASG shapes EHRA, we analyze, as an intermediate step, how the intensity of government health expenditure and the allocation of human resources for health play a role in FASG impacting EHRA. In our analysis, it is also found that the improvement of FASG can prompt subnational governments to increase the fiscal expenditure on medical and health. The FASG determines the level of supply of medical and health services to a certain extent (60), i.e., the fuller FASG, the more capable subnational governments are to improve the local medical and health situation by optimizing the fiscal expenditure structure expanding the coverage of medical and health resources (61), and gradually flattening the gap in medical and health services between regions and between urban and rural areas. Our study also points out that FASG can improve the equity of medical and health resources by optimizing AHRH. Human resources for health are the fundamental guarantee for the operation of the health system and an essential element in the allocation of medical and health resources (62). However, in China, the training of medical and health workers has always been a weak one, which depends on effective and sustainable government investment (63). The higher FASG means that the government has more fiscal resources and more vital fiscal capability to expand the quantity and quality of workers for health through education, training, and optimal allocation and ultimately promote the equity of the resources of the entire health system (64, 65). In other words, some regions are those where a large share of spending is financed with their revenues, which favors the increase in the accountability and motivation of subnational government for EHRA, who then provide more appropriate and more targeted services, improving the governance of the healthcare system (66). In summary, we believe that the higher FASG can at least improve EHRA through two channels: expanding medical and health expenditure and optimizing the allocation of medical and health human resources.

In the last part of the econometric analysis, we also analyzed and discussed the heterogeneity of the impact of FASG on EHRA. Consistent with our expectation, we observe that under different PGDP constraints, the promotion effect of FASG on EHRA is quite different. As the PGDP exceeds the first threshold value, the promotion effect decreases. Furthermore, when the second threshold value is exceeded, the corresponding estimated coefficient further decreases and becomes insignificant (only Jiangsu, Zhejiang, Guangdong, and other provinces meet in some years). This situation indicates that the uneven healthcare resource allocation in areas with more backward economic development is often more severe, and the subnational government's fiscal investment in rural, remote, or backward areas can obtain more short-term benefits, ultimately conducive to improving EHRA. From the perspective of PD, the promotion effect of FASG on EHRA is more evident in areas with lower PD, while the promotion effect is not significant in areas with higher PD. A reasonable explanation for this might be that there is often agglomeration in areas with high PD (67), which may derive and intensify the “siphon effect” of large cities on medical and health resources to a certain extent. Even if local governments invest a lot of fiscal funds in areas with scarce medical and health resources, the improvement of EHRA in the jurisdiction may still have little effect. Finally, from the perspective of DR, the difference from the test results of the two threshold variables mentioned above is that when DR exceeds the corresponding threshold value, the promotion effect of FASG on EHRA is magnified. In terms of reality, due to the large-scale movement of labor and population over the years, the aging of the population (68) and the problem of left-behind children in rural or remote areas (69, 70) are much more severe than in cities. Needless to say, it will greatly reinforce the pressure on child raising and elderly supporting in areas with weak economic and social development. Moreover, children and the elderly are often vulnerable groups in terms of health, and there is a greater demand for medical and health resources and services. Changes in DR and other demographic factors have forced subnational governments to allocate more medical and health resources to remote and underdeveloped areas to alleviate the demand pressure on medical resources caused by vulnerable groups and improve EHRA in the region. To a certain extent, our research helps to inspire and promote researchers in the field of public health to pay more attention to the fiscal capability and scale of subnational governments' investment in identifying the influencing factors of EHRA. In addition, for government departments, our research also has a certain guiding role in policy formulation. Specific measures include strengthening the primary responsibilities of subnational governments in promoting EHRA, building a stable mechanism for the investment and allocation of medical and health resources, exploring a practical path of “urban feeding back to the countryside” of medical and health resources, and making up for the shortcomings of medical and health care in grassroots areas and backward areas through infrastructure renewal and personnel team construction, which will help to alleviate further the problem of excessive agglomeration of medical and health resources in cities and developed areas. However, what we need to pay special attention to is that when subnational governments that are generally facing rapid growth of public debt (71) need to bear the continuous fiscal burden caused by the prevention and control of COVID-19, subnational governments should coordinate the use of limited financial funds, adhere to and strengthen support policies and investment in remote and underdeveloped areas, and be wary of the excessive absorption of fiscal funds by large cities in the province due to the need for epidemic prevention and control, to prevent the further exacerbation of the unequal degree of medical and health resources in the province.

Although compared with existing research, our research constructed a more reasonable EHRA index, introduced FASG as an essential institutional variable in the analysis, and offers valuable insights into the above conclusion after a series of rigorous and standardized econometric analyses. However, this study also has some limitations. Due to the severe lack of data in some provinces, only the data of 22 provinces (2011–2020) are included in our study. These provinces not included in the study have a common feature: their economic and social development is less developed. If data is available, whether the inclusion of other provinces in the study will challenge the conclusions of this study is an issue worthy of attention. Besides, this study only focuses on provincial-level problems rather than using more specific data from prefecture-level cities for research. Although constructing the EHRA index of prefecture-level cities requires detailed data from the next-level areas, which means that data collection will face lots of difficulties, further and additional studies will be needed to develop a full picture of the relationship between FASG and EHRA.

## Conclusion

As an essential gripper for realizing the “Healthy China” strategy, EHRA has been highly valued by governments at all levels, from the central government to the subnational government, and has gradually realized the transformation from a severe fundamental problem to a critical policy agenda. In our view, FASG, to a large extent, is associated with improved EHRA. On the one hand, this promotion effect can be achieved by increasing government medical and health expenditure and optimizing the allocation of human resources for health. On the other hand, the promotion effect of FASG on EHRA is constrained by economic and demographic conditions and exhibits specific nonlinear characteristics. In general, these conclusions are of enlightening significance for studying and solving real medical problems in China.

## Data availability statement

The datasets presented in this study can be found in online repositories. The names of the repository/repositories and accession number(s) can be found in the article/supplementary material.

## Author contributions

CY was the main person who designed the study, supervised all fieldwork, analyzed and interpreted the data and drafted the manuscript. DC and SY revised the manuscript. XL interpreted partial results. RW, YY, XK, YS, LX, and CT collected and analyzed partial data. All authors contributed to the article and approved the submitted version.

## Conflict of interest

The authors declare that the research was conducted in the absence of any commercial or financial relationships that could be construed as a potential conflict of interest.

## Publisher's note

All claims expressed in this article are solely those of the authors and do not necessarily represent those of their affiliated organizations, or those of the publisher, the editors and the reviewers. Any product that may be evaluated in this article, or claim that may be made by its manufacturer, is not guaranteed or endorsed by the publisher.

## Abbreviations

EHRA: equity in the allocation of health care resources
FASG: fiscal autonomy of subnational governments
IGHE: intensity of government health expenditure
AHRH: Allocation of human resources for health
PGDP: per capita GDP
PD: population density
TA: traffic accessibility
DR: dependency ratio
IR: illiteracy rate
FE: fixed effects
RE: random effects
2SLS: two-stage least squares.

## References

[1] Ostlin P Braveman P Dachs JN Dahlgren G Diderichsen F Harris E. Priorities for research to take forward the health equity policy agenda. Bull World Health Organ. (2005) 83:948–53. 16462988 PMC2626494

[2] Mills A Ataguba JE Akazili J Borghi J Garshong B Makawia S. Equity in financing and use of health care in Ghana, South Africa, and Tanzania: implications for paths to universal coverage. Lancet. (2012) 380:126–33. 10.1016/S0140-6736(12)60357-2 22591542

[3] Yu M He SJ Wu DX Zhu HP Webster C. Examining the multi-scalar unevenness of high-quality healthcare resources distribution in China. Int J Environ Res Public Health. (2019) 16:20. 10.3390/ijerph16162813 31394765 PMC6720903

[4] Braveman P. What is health equity: and how does a life-course approach take us further toward it? Matern Child Health J. (2014) 18:366–72. 10.1007/s10995-013-1226-9 23397099

[5] White-Means S Gaskin DJ Osmani AR. Intervention and public policy pathways to achieve health care equity. Environ Res Public Health. (2019) 16:2465. 10.3390/ijerph16142465 31373297 PMC6679008

[6] Love-Koh J Griffin S Kataika E Revill P Sibandze S Walker S. Methods to promote equity in health resource allocation in low- and middle-income countries: an overview. Global Health. (2020) 16:6. 10.1186/s12992-019-0537-z 31931823 PMC6958737

[7] Hosseinpoor AR Bergen N Schlotheuber A. Promoting health equity: WHO health inequality monitoring at global and national levels. Glob Health Action. (2015) 8:29034. 10.3402/gha.v8.29034 26387506 PMC4576419

[8] Yin CH He QS Liu YF Chen WQ Gao Y. Inequality of public health and its role in spatial accessibility to medical facilities in China. Appl Geogr. (2018) 92:50–62. 10.1016/j.apgeog.2018.01.011

[9] Rong PJ Zheng ZC Kwan MP Qin YC. Evaluation of the spatial equity of medical facilities based on improved potential model and map service API: a case study in Zhengzhou, China. Appl Geogr. (2020) 119:14. 10.1016/j.apgeog.2020.102192

[10] Weiss DJ Nelson A Vargas-Ruiz CA Gligoric K Bavadekar S Gabrilovich E. Global maps of travel time to healthcare facilities. Nat Med. (2020) 26:11. 10.1038/s41591-020-1059-1 32989313

[11] Yang L Sun L Wen L Zhang H Li C Hanson K. Financing strategies to improve essential public health equalization and its effects in China. Int J Equity Health. (2016) 15:194. 10.1186/s12939-016-0482-x 27905941 PMC5134004

[12] Zhang T Xu Y Ren J Sun L Liu C. Inequality in the distribution of health resources and health services in China: hospitals vs. primary care institutions. Int J Equity Health. (2017) 16:42. 10.1186/s12939-017-0543-9 28253876 PMC5335774

[13] Li Q Wei JJ Jiang FC Zhou GX Jiang RL Chen MJ. Equity and efficiency of health care resource allocation in Jiangsu Province, China. Int J Equity Health. (2020) 19:13. 10.1186/s12939-020-01320-2 33246458 PMC7694921

[14] Tao WJ Zeng Z Dang HX Li PY Chuong L Yue DH. Toward universal health coverage: achievements and challenges of 10 years of healthcare reform in China. BMJ Glob Health. (2020) 5:10. 10.1136/bmjgh-2019-002087 32257401 PMC7103842

[15] Yip WN Fu HQ Chen AT Zhai TM Jian WY Xu RM. 10 years of health-care reform in China: progress and gaps in Universal Health coverage. Lancet. (2019) 394:1192–204. 10.1016/S0140-6736(19)32136-1 31571602

[16] Qin XZ Hsieh CR. Economic growth and the geographic maldistribution of health care resources: Evidence from China, 1949–2010. China Econ Rev. (2014) 31:228–46. 10.1016/j.chieco.2014.09.010

[17] Chen J Lin ZC Li LA Li J Wang YY Pan Y. Ten years of China's new healthcare reform: a longitudinal study on changes in health resources. BMC Public Health. (2021) 21:13. 10.1186/s12889-021-12248-9 34903184 PMC8670033

[18] Chai KC Zhang YB Chang KC. Regional disparity of medical resources and its effect on mortality rates in China. Front Public Health. (2020) 8:8. 10.3389/fpubh.2020.00008 32117848 PMC7011092

[19] Yao H Zhan C Sha X. Current situation and distribution equality of public health resource in China. Arch Public Health. (2020) 78:86. 10.1186/s13690-020-00474-3 32983449 PMC7507592

[20] Chen YY Yin Z Xie Q. Suggestions to ameliorate the inequity in urban/rural allocation of healthcare resources in China. Int J Equity Health. (2014) 13:6. 10.1186/1475-9276-13-34 24884614 PMC4016733

[21] Artiga S Hinton E. Beyond health care: the role of social determinants in promoting health and health equity. Health. (2019) 20:1–13.25217354

[22] Newdick C Derrett S. Access, equity and the role of rights in health care. Health Care Anal. (2006) 14:157–68. 10.1007/s10728-006-0023-7 17214251

[23] Asante AD Zwi AB. Factors influencing resource allocation decisions and equity in the health system of Ghana. Public Health. (2009) 123:371–7. 10.1016/j.puhe.2009.02.006 19364613

[24] Zhang Y Wang Q Jiang T Wang J. Equity and efficiency of primary health care resource allocation in mainland China. Int J Equity Health. (2018) 17:12. 10.1186/s12939-018-0851-8 30208890 PMC6134520

[25] Ding JM Hu XJ Zhang XZ Shang L Yu M Chen HL. Equity and efficiency of medical service systems at the provincial level of China's mainland: a comparative study from 2009 to 2014. BMC Public Health. (2018) 18:14. 10.1186/s12889-018-5084-7 29402260 PMC5799902

[26] Lu C Zhang ZX Lan XT. Impact of China's referral reform on the equity and spatial accessibility of healthcare resources: a case study of Beijing. Soc Sci Med. (2019) 235:9. 10.1016/j.socscimed.2019.112386 31272079

[27] Kyriacou AP Roca-Sagales O. Local decentralization and the quality of public services in Europe. Soc Indic Res. (2019) 145:755–76. 10.1007/s11205-019-02113-z

[28] Thanh SD Nguyen CP Bui DT Binh NQ Van DTB. Spatial spillover effects of fiscal decentralization on governance and public administration quality. Reg Stud. (2022) 19:1–19.

[29] Tao Y Henry K Zou Q Zhong X. Methods for measuring horizontal equity in health resource allocation: a comparative study. Health Econ Rev. (2014) 4:10. 10.1186/s13561-014-0010-x 26054400 PMC4884040

[30] Dong E Xu J Sun X Xu T Zhang L Wang T. Differences in regional distribution and inequality in health-resource allocation on institutions, beds, and workforce: a longitudinal study in China. Arch Public Health. (2021) 79:78. 10.1186/s13690-021-00597-1 34001268 PMC8130126

[31] Isabel C Paula V. Geographic distribution of physicians in Portugal. Eur J Health Econ. (2010) 11:383–93. 10.1007/s10198-009-0208-8 20012127

[32] Horev T Pesis-Katz I Mukamel DB. Trends in geographic disparities in allocation of health care resources in the US. Health Policy. (2004) 68:223–32. 10.1016/j.healthpol.2003.09.011 15063021

[33] Chang B Yang Y Buitrago Leon GA Lu Y. Effect of collaborative governance on medical and nursing service combination: an evaluation based on delphi and entropy method. Healthcare. (2021) 9:1456. 10.3390/healthcare9111456 34828502 PMC8622114

[34] Satoła Ł Standar A Kozera A. Financial autonomy of local government units: Evidence from Polish rural municipalities. Lex Localis. (2019) 17:321–42. 10.4335/17.2.321-342(2019)

[35] Kapidani M. A comparative analysis of local government financial autonomy in Albania. J Bus Econ Finance. (2018) 7:1–9. 10.17261/Pressacademia.2018.790

[36] Fang H Zhang J. Reassessment of the incentive effects of fiscal centralization: a grabbing hand or a helping hand? J Manage World. (2014) 2:21–31. 10.19744/j.cnki.11-1235/f.2014.02.004

[37] Ding Y McQuoid A Karayalcin C. Fiscal decentralization, fiscal reform, and economic growth in china. China Econ Rev. (2019) 53:152–67. 10.1016/j.chieco.2018.08.005

[38] Zhu D Yuan Y Gao P. The nonlinear effect of fiscal decentralization on efficiency of local the nonlinear effect of fiscal decentralization on efficiency of local financial expenditure on medicine and health. Finance Econ. (2020) 8:118–32.

[39] Liu S. Local government's fiscal self-sufficiency and livelihood expenditure bias:a theoretical analysis and empirical test. Chin Public Administrat. (2021) 9:110–117. 10.19735/j.issn.1006-0863.2021.09.15

[40] Lin BQ Zhou YC. Understanding the institutional logic of urban environmental pollution in China: Evidence from fiscal autonomy. Process Saf Environ Protect. (2022) 164:57–66. 10.1016/j.psep.2022.06.005

[41] Hu FZY Qian JW. Land-based finance, fiscal autonomy and land supply for affordable housing in urban China: a prefecture-level analysis. Land Use Pol. (2017) 69:454–60. 10.1016/j.landusepol.2017.09.050

[42] Jin H Li BY Jakovljevic M. How China controls the Covid-19 epidemic through public health expenditure and policy? J Med Econ. (2022) 25:437–49. 10.1080/13696998.2022.2054202 35289700

[43] Micah AE Solorio J Stutzman H Zhao YX Tsakalos G Dieleman JL. Development assistance for human resources for health, 1990–2020. Hum Resour Health. (2022) 20:12. 10.1186/s12960-022-00744-x 35689228 PMC9187148

[44] Imai K Kim IS. On the use of two-way fixed effects regression models for causal inference with panel data. Polit Anal. (2021) 29:405–15. 10.1017/pan.2020.33

[45] Hansen BE. Threshold effects in non-dynamic panels: Estimation, testing, and inference. J Econom. (1999) 93:345–68. 10.1016/S0304-4076(99)00025-1

[46] Wang KM. Health care expenditure and economic growth: Quantile panel-type analysis. Econ Model. (2011) 28:1536–49. 10.1016/j.econmod.2011.02.008

[47] Hartwig J. What drives health care expenditure?—Baumol's model of ‘unbalanced growth'revisited. J Health Econ. (2008) 27:603–23. 10.1016/j.jhealeco.2007.05.006 18164773

[48] Feltenstein A Iwata S. Decentralization and macroeconomic performance in China: regional autonomy has its costs. J Dev Econ. (2005) 76:481–501. 10.1016/j.jdeveco.2004.01.004

[49] Yip WC-M Hsiao WC Chen W Hu S Ma J Maynard A. Early appraisal of China's huge and complex health care reforms. Health Care Policy East Asia World Sci Ref Volume 1 Health Care Syst Reform Policy Res China. (2020) 1:51–83. 10.1142/9789813236134_000422386036

[50] Kutzin J. Health financing for universal coverage and health system performance: concepts and implications for policy. Bull World Health Organ. (2013) 91:602–11. 10.2471/BLT.12.113985 23940408 PMC3738310

[51] Anselmi L Lagarde M Hanson K. Going beyond horizontal equity: an analysis of health expenditure allocation across geographic areas in Mozambique. Soc Sci Med. (2015) 130:216–24. 10.1016/j.socscimed.2015.02.012 25721333

[52] Hansen BE. Sample splitting and threshold estimation. Econometrica. (2000) 68:575–603. 10.1111/1468-0262.00124

[53] Dong EH Liu SP Chen MJ Wang HM Chen LW Xu T. Differences in regional distribution and inequality in health-resource allocation at hospital and primary health center levels: a longitudinal study in Shanghai, China. BMJ Open. (2020) 10:e035635. 10.1136/bmjopen-2019-035635 32690509 PMC7371131

[54] Wang Y Feng L. Resource allocation effects and improvements of quasi- marketization reform in China's health and medical industry. China Ind Econ. (2007) 24–31.

[55] Jiang W Wang W. The solution of the problems of the accessibility and affordability of health care in China: from the perspective of supply-side structural reform. Truth Seeking. (2017) 8:55–66.

[56] Xin C Li J YANG C. Research on regional difference and spatial convergence of medical and health service supply in China. Chin J Popul Sci. (2020) 1:65–77.

[57] Brixi H Mu Y Targa B Hipgrave D. Engaging sub-national governments in addressing health equities: challenges and opportunities in China's health system reform. Health Policy Plan. (2013) 28:809–24. 10.1093/heapol/czs120 23221008

[58] Bloom G. Building institutions for an effective health system: Lessons from China's experience with rural health reform. Soc Sci Med. (2011) 72:1302–9. 10.1016/j.socscimed.2011.02.017 21439699

[59] Long XL Ji X. Economic growth quality, environmental sustainability, and social welfare in China—provincial assessment based on genuine progress indicator (GPI). Ecol Econ. (2019) 159:157–76. 10.1016/j.ecolecon.2019.01.002

[60] Wang MY Tao CH. Research on the efficiency of local government health expenditure in China and its spatial spillover effect. Sustainability. (2019) 11:2469. 10.3390/su1109246931336960

[61] Rubio DJ. The impact of decentralization of health services on health outcomes: evidence from Canada. Appl Econ. (2011) 43:3907–17. 10.1080/00036841003742579

[62] Chen L Evans T Anand S Boufford JI Brown H Chowdhury M. Human resources for health: overcoming the crisis. Lancet. (2004) 364:1984–90. 10.1016/S0140-6736(04)17482-5 15567015

[63] Deng F Lv JH Wang HL Gao JM Zhou ZL. Expanding public health in China: an empirical analysis of healthcare inputs and outputs. Public Health. (2017) 142:73–84. 10.1016/j.puhe.2016.10.007 28057203

[64] Anand S Fan VY Zhang J Zhang L Ke Y Dong Z. China's human resources for health: quantity, quality, and distribution. Lancet. (2008) 372:1774–81. 10.1016/S0140-6736(08)61363-X 18930528

[65] Anand S Barnighausen T. Human resources and health outcomes: cross-country econometric study. Lancet. (2004) 364:1603–9. 10.1016/S0140-6736(04)17313-3 15519630

[66] Di Novi C Piacenza M Robone S Turati G. Does fiscal decentralization affect regional disparities in health? Quasi-experimental evidence from Italy. Regional Sci. Urban Econ. (2019) 78:103465. 10.1016/j.regsciurbeco.2019.103465

[67] Du M Liu C. Agglomeration effect, population mobility and urban growth. Populat. Econ. (2014) 6:44–56. 10.3969/j.issn.1000-4149.2014.06.005

[68] Tong Y. Changes and challenges of labor supply in China in the context of population aging. Populat. Res. (2014) 38:52–60.

[69] Li Q Liu G Zang W. The health of left-behind children in rural China. China Econ. Rev. (2015) 36:367–76. 10.1016/j.chieco.2015.04.004

[70] Jingzhong Y. Left-behind children: the social price of China's economic boom. J Peasant Stud. (2011) 38:613–50. 10.1080/03066150.2011.582946 21744548

[71] Li T Du T. Vertical fiscal imbalance, transfer payments, and fiscal sustainability of local governments in China. Int Rev Econ Finance. (2021) 74:392–404. 10.1016/j.iref.2021.03.019

